# Macrophage‐derived exosomes mediate silica‐induced pulmonary fibrosis by activating fibroblast in an endoplasmic reticulum stress‐dependent manner

**DOI:** 10.1111/jcmm.16524

**Published:** 2021-04-08

**Authors:** Xiaofeng Qin, Xiaofang Lin, Lang Liu, Ying Li, Xiang Li, Zhenghao Deng, Huiping Chen, Hui Chen, Zhiyuan Niu, Zisheng Li, Yongbin Hu

**Affiliations:** ^1^ Department of Pathology School of Basic Medical Science Central South University Changsha China; ^2^ Department of Occupational Diseases Hunan Prevention and Treatment Institute for Occupational Diseases Changsha China; ^3^ Department of Pathology Xiangya Hospital Central South University Changsha China

**Keywords:** ER stress, exosomes, fibroblasts, macrophages, silicosis

## Abstract

Macrophages play a key role in silicosis, and exosomes are potent mediators of intercellular communication. This suggests that macrophage‐derived exosomes have a potential contribution to the pathogenesis of silicosis. To investigate whether macrophage‐derived exosomes promote or inhibit lung fibrosis, in vitro, silica‐exposed macrophage‐derived exosomes (SiO_2_‐Exos) were collected and cocultured with fibroblasts. The expression of collagen I and α‐SMA was evaluated. Furthermore, the endoplasmic reticulum (ER) stress markers BIP, XBP1s and *P*‐eIF2α were assessed after treatment with or without the ER stress inhibitor 4‐PBA. In vivo, mice were pre‐treated with the exosome secretion inhibitor GW4869 prior to silica exposure. After sacrifice, lung tissues were histologically examined, and the expression of proinflammatory cytokines (TNF‐α, IL‐1β and IL‐6) in bronchoalveolar lavage fluid (BALF) was measured. The results showed that the expression of collagen I and α‐SMA was up‐regulated after treatment with SiO_2_‐Exos, accompanied by increased expression of BIP, XBP1s and *P*‐eIF2α. Pre‐treatment with 4‐PBA reversed this effect. More importantly, an in vivo study demonstrated that pre‐treatment with GW4869 decreased lung fibrosis and the expression of TNF‐α, IL‐1β and IL‐6 in BALF. These results suggested that SiO_2_‐Exos are profibrogenic and that the facilitating effect is dependent on ER stress.

## 1 INTRODUCTION

Silicosis is a traditional occupational disease with unfavourable prognosis, and it is usually caused by prolonged inhalation of free silica dust (SiO_2_). The characteristics of silicosis include extensive silicon nodule formation, chronic inflammation, aberrant fibroblast activation and excessive extracellular matrix (ECM) deposition, which eventually lead to abnormal lung tissue repair and irreversible pulmonary failure.^1^, ^2^ Recent epidemiological work has shown that silicosis is still one of the severest occupational diseases around the world, especially in low‐ and middle‐income countries.^3^ China has the most patients with silicosis, with more than 873 000 cases recorded through the end of 2018. According to a document from the National Health Commission of the People's Republic of China, China had 15 898 new cases of pneumoconiosis in 2019. The reported cases of occupational respiratory diseases accounted for 82.08% of the total reported cases of occupational diseases.^4^ Despite the multiple efforts made in recent years, treatments for silicosis are still not satisfactory.^2^, ^3^ Therefore, we need to further expand the understanding of silicosis at the cellular and molecular levels and improve therapies for this disease.

Alveolar macrophages and fibroblasts play crucial roles in the process of silicosis. Alveolar macrophages initially recognize and capture inhaled silica particles, and then, the process of inflammasome activation proceeds.^3^, ^5^, ^6^ Various proinflammatory and profibrotic factors are released and activated, such as transforming growth factor‐β (TGF‐β), interleukin (IL)‐6, IL‐1β and tumour necrosis factor‐α (TNF‐α).^2^, ^7^, ^8^, ^9^, ^10^ Then, these cytokines trigger the differentiation of fibroblasts into activated myofibroblasts, promote fibroblast recruitment and proliferation, and result in excessive accumulation of collagen fibres, eventually leading to pulmonary fibrosis.^2^ To date, the crosstalk between macrophages and fibroblasts and how these cells communicate with each other to deal with stress from SiO_2_ exposure in the pathogenesis of silicosis have not been fully elucidated. In addition, non‐specific anti‐inflammatory or antifibrotic therapy has not achieved a good therapeutic effect in clinical settings.^1^, ^2^

Exosomes are membrane‐like vesicles with a diameter of 30‐150 nm, and they are considered mediators of both local and distant intercellular communication. Exosomes contribute to biological processes by transporting a variety of cell‐ and cell‐state‐specific cargo, including nucleic acids, lipids and proteins.^11^ Mounting evidence has demonstrated that exosomes widely participate in tumour progression, immune responses, heart failure, diabetes, etc^12^, ^13^, ^14^ In addition, recent studies have revealed the potential contribution of exosomes to the pathogenesis of chronic lung diseases.^15^, ^16^ For example, the secretion level of exosomes into bronchoalveolar lavage fluid (BALF) is increased in human and experimental lung fibrosis, and exosomes found in increased amounts function as carriers of protein/miRNA that contribute to idiopathic pulmonary fibrosis (IPF) pathogenesis.^17^, ^18^ However, the secretion and potential contribution of alveolar macrophage‐derived exosomes in silicosis remain largely unexplored.

Cellular dysfunctions caused by disruption of endoplasmic reticulum (ER) homeostasis contribute to the development of fibrotic diseases.^19^, ^20^, ^21^ The ER is a special organelle where proteins are folded, processed and quality‐controlled to maintain proteostasis. ER dysfunction can give rise to the accumulation of unfolded or misfolded proteins, resulting in homeostasis imbalance, a condition termed ER stress, that triggers unfolded protein response (UPR, a signalling cascade activated in response to ER stress) hyperactivation.^22^ The UPR is mainly composed of three signalling pathways: (1) inositol‐requiring enzyme‐1α (IRE1α)‐XBP1s, (2) protein kinase RNA (PKR)‐like ER kinase (PERK)‐*P*‐eIF2α and (3) activating transcription factor 6 (ATF6).^23^ The aim of UPR activation is to restore proteostasis; however, prolonged or overloaded ER stress may result in cell death.^24^ Mounting evidence indicates that ER stress is involved in pulmonary fibrosis by regulating myofibroblast transdifferentiation, alveolar epithelial cell apoptosis, M2 macrophage polarization and epithelial‐mesenchymal transition (EMT).^22^ Inhibition of ER stress can effectively suppress TGF‐β1‐induced fibroblast activation and tissue fibrosis.^25^ Additionally, a recent study showed that HeLa cell‐derived exosomes disrupt vascular integrity by activating ER stress in vascular endothelial cells.^26^ Consequently, we hypothesized that exosomes derived from SiO_2_‐exposed macrophages (SiO_2_‐Exos) may promote myofibroblast activation through ER stress.

In the present study, we revealed that SiO_2_‐Exos could promote myofibroblast differentiation, proliferation and migration, and that inhibition of ER stress could reverse the fibrotic phenotype of activated myofibroblasts. Inhibition of exosome generation dampened SiO_2_‐induced lung fibrosis and the inflammatory response in mice.

## 2 MATERIALS AND METHODS

### 2.1 Cell lines and cell culture

The mouse macrophage cell line RAW264.7, mouse embryonic fibroblast cell line NIH‐3T3, human monocyte leukaemia cell line THP‐1 and human embryonic fibroblast cell line HFL1 were purchased from the China Center for Type Culture Collection (CCTCC). HFL1 cells were cultured in Ham's F‐12K (Kaighn's) medium (F12k; Gibco) supplemented with 10% foetal bovine serum (FBS; BI, Kibbutz Beit‐Haemek). RAW264.7 and NIH3T3 cells were cultured in Dulbecco's modified Eagle's medium (DMEM; Gibco) supplemented with 10% FBS. THP‐1 cells were cultured in Roswell Park Memorial Institute (RPMI)‐1640 medium (BI, Kibbutz Beit‐Haemek, Israel) supplemented with 10% FBS. All the cells were cultured at 37°C with 5% CO_2_. To perform a wound closure assay, cells were cultured in growth medium without FBS. THP‐1 cells were treated with phorbol 12‐myristate 13‐acetate (PMA; 100 ng/mL, Sigma‐Aldrich, Merck KGaA,) for 24 hours to induce differentiation into an adherent macrophage phenotype before SiO_2_ exposure. Before administration, silicon dioxide (SiO_2_; S5631, 1‐5 µm, Sigma‐Aldrich, Merck KGaA,) was packaged in tinfoil and baked overnight (180°C, 16 hours) for sterilization and inactivation of any endotoxin contamination. In this study, the SiO_2_ dosage was 200 μg/mL. The conditioned medium of macrophages was harvested after treatment with SiO_2_ for 48 hours. For an ER stress inhibition assay, fibroblasts were pre‐treated with 4‐phenylbutyric acid (4‐PBA) (500 μmol/L, Sigma‐Aldrich, Merck KGaA,) for 4 hours in growth medium without FBS. After washing with sterile phosphate‐buffered saline (PBS) 3 times, these fibroblasts were treated with SiO_2_‐Exos +4‐PBA (500 μmol/L) for different time points in growth medium supplemented with 10% FBS.

### 2.2 Exosome isolation, identification and treatment

Exosomes were isolated from conditioned medium of RAW264.7 or THP‐1 cells by differential centrifugation (Beckman Coulter). RAW264.7 and THP‐1 cells were cultured in growth medium with 10% exosome‐depleted FBS, and the cell culture supernatant (SN) was collected after 48 hours of culture. The collected SN was centrifuged at 3 000 × *g* at 4°C for 10 minutes to remove cells and cellular debris and then at 20 000 × *g* at 4°C for 25 minutes to remove microvesicles. Vesicles smaller than 200 nm were collected by centrifugation at 110 000 × *g* at 4°C for 120 minutes after filtration with a 0.22 μm filter (Millipore, MA). The precipitate was resuspended in ice‐cold sterile PBS, and the mixture was ultracentrifuged at 110 000 × *g* at 4°C for 120 minutes to remove contaminated proteins. Finally, the exosome pellets were resuspended in specific resuspension buffers according to different objectives. These exosomes could be used immediately or stored at −80°C. The number of exosomes was determined by measuring the total protein of exosomes by a Micro‐BCA assay (Pierce). To verify the exosomes, the expression of the extracellular vesicle‐related protein markers CD63 (1:1000; ab134045; Abcam) and HSP70 (heat shock protein 70; 1:1 000; ab2787; Abcam) and the exosome‐specific marker TSG101 (tumour susceptibility gene 101; 1:1 000; 14497‐1‐AP; Proteintech) was detected by Western blot analysis. For transmission electron microscopy (TEM; FEI) analysis, exosomes were prefixed with 1% glutaraldehyde, followed by negative staining with a negative‐staining solution (4% uranyl acetate (pH 4) + 2% methylcellulose). Then, the preparation could be viewed and imaged by TEM. The range of size distribution of exosomes was confirmed by nanoparticle tracking analysis (NTA; Zetasizer Nano ZS, Malvern Instruments, Worcestershire). For cell treatment, 1 × 10^5^ recipient cells were treated with 50 μg of exosomes at different time points.

### 2.3 Trafficking analysis of exosomes

To dynamically trace exosomes, the exosomes were labelled with a PKH26 fluorescent kit (Sigma‐Aldrich, Merck KGaA). First, according to the manufacturer's directions, the purified exosomes were incubated with PKH26 dye. Next, the mixture was diluted in ice‐cold PBS and then centrifuged at 110 000 × *g* at 4°C for 90 minutes to collect the PKH26‐labelled exosome pellets, which were resuspended in 100 μL of sterile PBS and then added to coculture with HFL1 cells in complete growth medium for 24 hours. After washing 3 times with PBS, an inverted fluorescence microscope (Olympus) was used to detect the appearance of red fluorescence in HFL1 cells.

### 2.4 Exosome secretion inhibition assay

To inhibit the secretion of exosomes, RAW264.7 and THP‐1 cells were pre‐treated with the exosome secretion inhibitor GW4869 (10 μmol/L, Cayman Chemical)^27^ 24 hours prior to SiO_2_ exposure. RAW264.7 and THP‐1 cells were then treated with SiO_2_ (200 μg/mL) + GW4869 (10 μmol/L) for 48 hours and the cell culture SN was harvested.

### 2.5 Western blot analysis

Cell lysates and exosomal lysates were subjected to SDS‐PAGE. Western blot analyses were performed by using anti‐collagen I (1:1 000; 14695‐1‐AP; Proteintech), anti‐α‐smooth muscle actin (SMA) (1:1 000; 14395‐1‐AP; Proteintech), anti‐glyceraldehyde‐3‐phosphate dehydrogenase (GAPDH) (1:1 000; 14497‐1‐AP; Proteintech), anti‐immunoglobulin heavy chain binding protein (BIP) (1:1 000; 11587‐1‐AP; Proteintech), anti‐spliced X‐box binding protein 1 (XBP1s) (1:1 000; #12782; Cell Signaling Technology), and anti‐phospho eukaryotic initiation factor 2 (eIF2) α (*P*‐eIF2α) (1:1 000; #3398; Cell Signaling Technology) antibodies and corresponding HRP‐conjugated secondary antibodies. A chemiluminescent system (Amersham ECL Plus) was used for detection.

### 2.6 Quantitative RT‐PCR analysis of mRNA

Following the manufacturer's protocols, Trizol reagent (Thermo Scientific) was used to extract total RNA, and then, a NanoDrop 2000 spectrophotometer (Thermo Scientific) was used to evaluate RNA quality and quantity. Total RNA was reverse transcribed using a cDNA synthesis kit (Genecopoeia), and then, an ABI‐7500 instrument (Applied Biosystems; Thermo Fisher Scientific Inc Waltham) was used to perform real‐time PCR. The expression levels of collagen I and α‐SMA were normalized to GAPDH. The primers used in the reactions are listed below: mouse collagen I 5ʹGCTCCTCTTAGGGGCCACT3ʹ, 3ʹCCACGTCTCACCATTGGGG5ʹ; mouse α‐SMA 5ʹGTCCCAGACATCAGGGAGTAA3ʹ, 3ʹTCGGATACTTCAGCGTCAGGA5ʹ; mouse GAPDH 5ʹAGGTCGGTGTGAACGGATTTGʹ, 3ʹTGTAGACCATGTAGTTGAGGTCAʹ; human collagen I 5ʹCTCCGGCTCCTGCTCCTCTTAG3ʹ, 3ʹGGCAGTTCTTGGTCTCGTCACAG5ʹ, human GAPDH 5ʹGGCACCGTCAAGGCTGAGAACʹ, 3ʹTGCAGGAGGCATTGCTGATGATCʹ; and human α‐SMA 5ʹCAACGTGGAGCGCAGTGGTC3ʹ, 3ʹCAAGGCAGTGCTGTCCTCTTCTTC5ʹ.

### 2.7 Proliferation and a wound closure assay

Following the manufacturer's instructions, cell proliferation was detected by Cell Counting Kit‐8 (CCK‐8 Kit; Dojindo Molecular Technologies). Cell migration was assessed by a wound closure assay in a 2D culture system. 1 × 10^5^ HFL1 or NIH3T3 cells were inoculated into 24‐well tissue culture plates. When the cells were approximately 70%‐80% confluent, a cross‐shaped scratch was carefully drawn in each well using a sterile 200‐μL pipette tip. Each well was then washed 3 times to remove all the detached cells. Digital images were captured at different time points (0, 12 or 24 hours), and ImageJ software (National Institutes of Health) was used to quantitatively evaluate the gap width.

### 2.8 Animal model

The design and methods of the research are in accordance with the requirements of related regulations and procedures (such as the National Institutes of Health Guide for the Care and Use of Laboratory Animals) as well as ethical principles. Six‐week‐old male C57BL/6 mice were randomly divided into three groups (control, SiO_2_ and SiO_2_ + GW4869). The silicosis model was induced by intratracheal injection of silica (100 mg/kg bodyweight) that was suspended in 20 μL of sterile PBS. The control group received the same volume of sterile PBS. The animals were killed 28 days after silica exposure, and the lungs and BALF were collected for further analysis. For administration of an exosome secretion inhibitor, GW4869 (2.5 μg/g of bodyweight per day) was dissolved in PBS and pre‐administered intraperitoneally to each animal for 7 days before silica exposure. GW4869 was continuously administered to the mice until they were killed at 35 days for further study.

### 2.9 Haematoxylin and eosin, Masson's trichrome and immunohistochemical staining

Lungs were fixed overnight with 4% paraformaldehyde at a constant pressure to ensure that the lungs were totally submerged, and then, the lungs were dehydrated and embedded in paraffin. The sections were deparaffinized and stained with Masson's trichrome or haematoxylin and eosin (H&E) (Sigma‐Aldrich, Merck KGaA). Immunohistochemical staining was performed with an anti‐α‐SMA antibody (1:200; 14395‐1‐AP; Proteintech) as described previously.^28^ Digital images of the area of positive staining were captured by using a light microscope attached to an image‐analysis system (K‐Viewer, KFBIO).

### 2.10 Statistics

GraphPad Prism 5 (GraphPad Software) was used for statistical analysis. Numerical data were compared using Student's *t* test (unpaired, two‐tailed) or two‐way ANOVA, and the statistical significance was set at *P* < .05.

## 3 RESULTS

### 3.1 Exosome secretion is increased in the cell culture supernatant of RAW264.7 cells treated with SiO_2_

**FIGURE 1:**
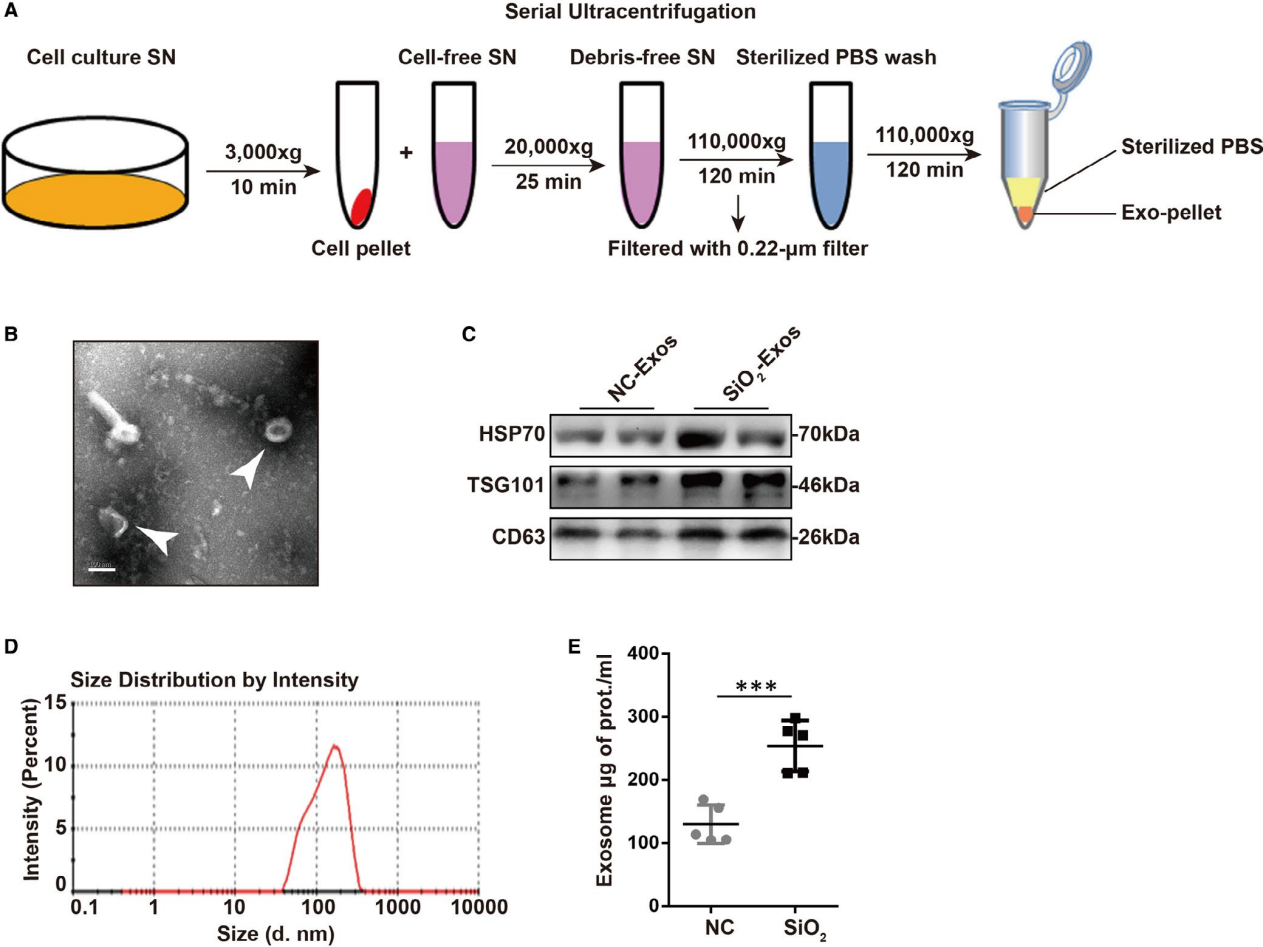
Exosome secretion is increased in the cell culture supernatant of RAW264.7 cells treated with silica (SiO_2_). (A) Scheme of the protocol used for the isolation of exosomes from the cell culture supernatant (SN). Abbreviations: exosomes: Exos; SN: cell culture supernatant; exosome pellet: Exo‐pellet. (B) A representative image of exosomes isolated from the SN of RAW264.7 cells treated with SiO_2_ taken with a transmission electron microscope. The SN was collected at 48 h post‐stimulation. The white arrows indicate exosomes. Scale bar: 100 nm. (C) The expression levels of the exosome‐related markers HSP70, TSG101 and CD63 were detected by Western blot analysis. Exosomes were isolated from the SN of blank control‐ (NC‐Exos) or SiO_2_‐treated (SiO_2_‐Exos) RAW264.7 cells (n = 2 per group). (D) Histogram showing the size distribution of exosomes analysed by nanoparticle tracking analysis (NTA). (E) Quantification of the total protein in exosomes isolated from the SN of vehicle‐ or SiO_2_‐treated RAW264.7 cells (n = 5 per group). ***P < .001, Student's *t* test

A recent study indicated that the secretion of exosomes into BALF is up‐regulated in bleomycin‐induced lung fibrosis or idiopathic pulmonary fibrosis,^17^ and circulating endothelial microparticle levels are increased in chronic obstructive pulmonary disorder (COPD) patients.^29^ Hence, we first asked whether the secretion level of exosomes is altered in SiO_2_‐induced pulmonary fibrosis. For this purpose, we isolated and characterized exosomes from the cell culture supernatant of RAW264.7 cells treated with SiO_2_ or a blank control (Figure 1A). The supernatant was collected from RAW264.7 cells after SiO_2_ or vehicle administration for 48 hours. Morphological assessment of exosomes by TEM revealed the presence of exosomes (cup‐shaped vesicles with a diameter of 30‐150 nm; white arrows) (Figure 1B). We further found enriched expression of the exosome‐specific marker TSG101 and the specific endosomal markers CD63 and HSP70 in purified exosomes (Figure 1C). The expression levels of HSP70, TSG101 and CD63 were increased in SiO_2_‐Exos compared to exosomes from control‐treated macrophages (NC‐Exos), suggesting a potential increase in exosome secretion with SiO_2_‐exposure. Next, we detected the size distribution of the purified exosomes by NTA (Figure 1D), which showed that the sizes of exosomes were mostly between 30 and 200 nm (peak: l44.5 nm). In addition, we found that the total protein content of SiO_2_‐Exos increased significantly compared with NC‐Exos (Figure 1E, exosome total μg of protein/mL: NC 129.8 ± 13.63, SiO_2_ 253.7 ± 17.92, *P* = .0006).

### 3.2 SiO_2_‐Exos promote myofibroblast differentiation in vitro

**FIGURE 2:**
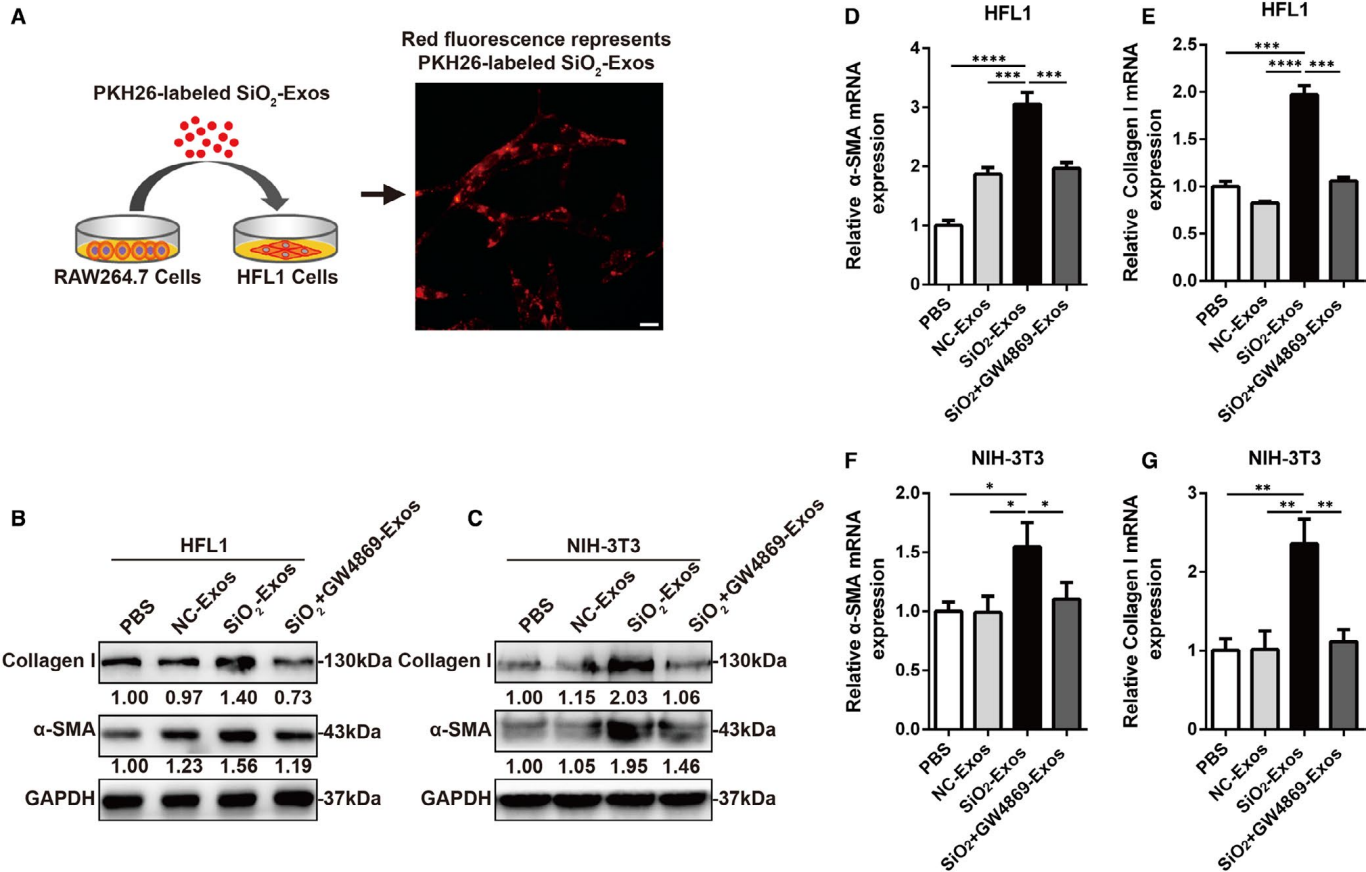
Macrophage‐derived exosomes modulate myofibroblast differentiation in vitro. (A) Exosomes derived from RAW264.7 cells were labelled with PKH26 dye and then incubated with HFL1 cells for 24 h. Scale bar: 20 μm. (B, D‐E) Western blot (B) and RT‐qPCR (D‐E) analyses of collagen I, α‐SMA and GAPDH in HFL1 cells treated with PBS, NC‐Exos, SiO_2_‐Exos or SiO_2_ + GW4869‐Exos were performed. These exosomes were derived from THP‐1 cells. (C, F‐G) Western blot (C) and RT‐qPCR (F‐G) analyses of collagen I, α‐SMA and GAPDH in NIH‐3T3 cells incubated with PBS, NC‐Exos, SiO_2_‐Exos or SiO_2_ + GW4869‐Exos were performed. These exosomes were derived from RAW264.7 cells. Student's *t* test; *P < .05, **P < .01

Previous studies have shown that the activation of macrophage induced by SiO_2_ initiates pulmonary fibrosis in silicosis, followed by myofibroblast activation and aberrant collagen deposition.^2^, ^3^, ^30^ One of the main mechanisms of myofibroblast activation is the phenotypic transdifferentiation of fibroblasts into myofibroblasts by which myofibroblasts arise and accumulate in lung tissue.^31^ Myofibroblasts are characterized by high expression of the myocyte‐specific isoform α‐SMA, and increased synthesis and secretion of ECM components (such as collagen I, III and V). Inhibition of myofibroblast differentiation and proliferation can effectively suppress the development of pulmonary fibrosis.^32^ Additionally, recent studies have indicated that exosomes play a crucial role in fibrotic diseases as carriers of intercellular communication.^15^, ^16^ The number of exosomes in the BALF of IPF patients is increased, and these exosomes promote lung fibroblast proliferation.^17^ Given that high secretion of exosomes is related to SiO_2_‐induced pulmonary fibrosis, we explored whether exosomes derived from macrophages mediate myofibroblast activation. First, we investigated whether macrophage‐secreted exosomes can be ingested by fibroblasts. SiO_2_‐Exos were labelled with the red fluorescent dye PKH26 (Figure 2A), and then, these exosomes were incubated with HFL1 cells for 24 hours. The red fluorescence in HFL1 cells showed that these exosomes had been effectively phagocytized by the cells (Figure 2A). These results indicate that exosomes derived from macrophages can be ingested by fibroblasts.

Next, we evaluated whether exosomes derived from macrophages can induce myofibroblast differentiation. HFL1 cells and NIH‐3T3 cells were treated with PBS, NC‐Exos, SiO_2_‐Exos or exosomes from macrophages treated with SiO_2_+GW4869 (SiO_2_+GW4869‐Exos). Notably, treatment with SiO_2_‐Exos resulted in significantly increased expression of collagen I and α‐SMA in HFL1 cells (Figure 2B) and NIH‐3T3 cells (Figure 2C), and these effects were attenuated by pre‐treatment of macrophages with GW4869 (Figure 2B,C), which inhibits exosome generation.^27^ Consistent with these observations, the mRNA expression levels of collagen I and α‐SMA were also increased in HFL1 cells (Figure 2D,E) and NIH‐3T3 cells (Figure 2F,G) treated with SiO_2_‐Exos, and these effects could be reversed by GW4869 pre‐treatment.

### 3.3 SiO_2_‐Exos promote myofibroblast proliferation and migration

**FIGURE 3:**
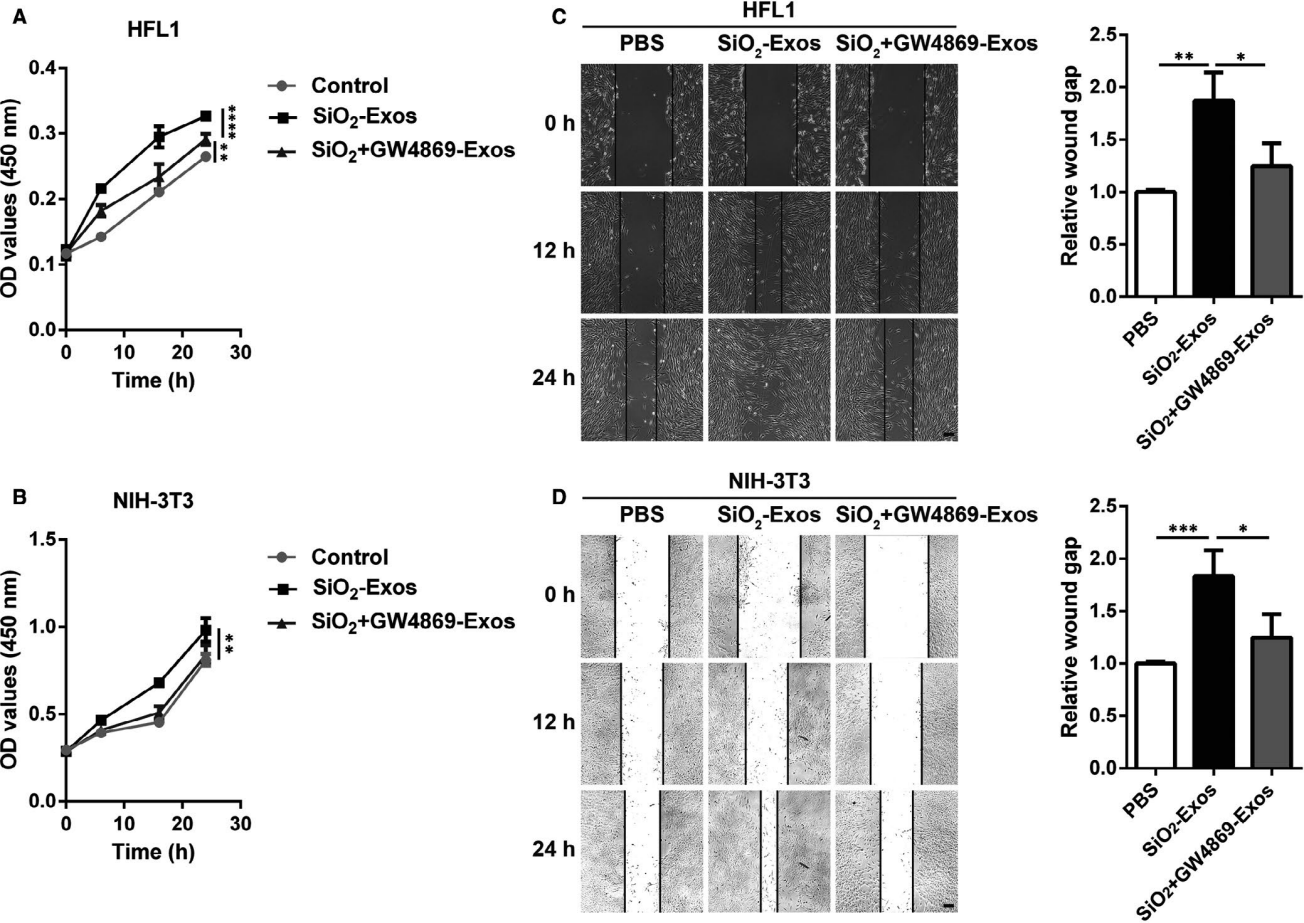
SiO_2_‐Exos induce lung fibroblast proliferation and migration (A‐B) CCK‐8 assay was used to evaluate the viability of HFL1 cells (A) and NIH‐3T3 cells (B) treated with various exosomes (SiO_2_‐Exos or SiO_2_ + GW4869‐Exos). Two‐way ANOVA; **P < .01, ***P < .001, n.s: not significant. (C‐D) The migration of HFL1 cells (C) and NIH‐3T3 cells (D) was assessed by wound closure assay. Scale bar: 100 μm. Student's *t* test; *P < .05, **P < .01, ***P < .001, n.s: not significant

Activated myofibroblasts proliferate and migrate to form fibrotic foci and produce excessive ECM components, which are characteristics of fibrotic diseases.^33^, ^34^ In addition, McBride et al ^35^ found that mesenchymal stem cell (MSC)‐derived exosomes could promote dermal fibroblast proliferation. Therefore, we next explored whether SiO_2_‐Exos modulate myofibroblast proliferation and migration. SiO_2_‐Exos or SiO_2_ + GW4869‐Exos were used to treat fibroblasts, and then, fibroblast proliferation and migration were measured. The results showed that SiO_2_‐Exos promoted HFL1 (Figure 3A) and NIH‐3T3 (Figure 3B) cell proliferation. Consistent with these observations, treatment with SiO_2_‐Exos significantly promoted the migration of HFL1 cells (Figure 3C) and NIH‐3T3 cells (Figure 3D), and these effects were attenuated by pre‐treatment of macrophages with GW4869.

### 3.4 Inhibition of exosome secretion blocks SiO_2_‐induced proinflammatory cytokine production and pulmonary fibrosis in mice

**FIGURE 4:**
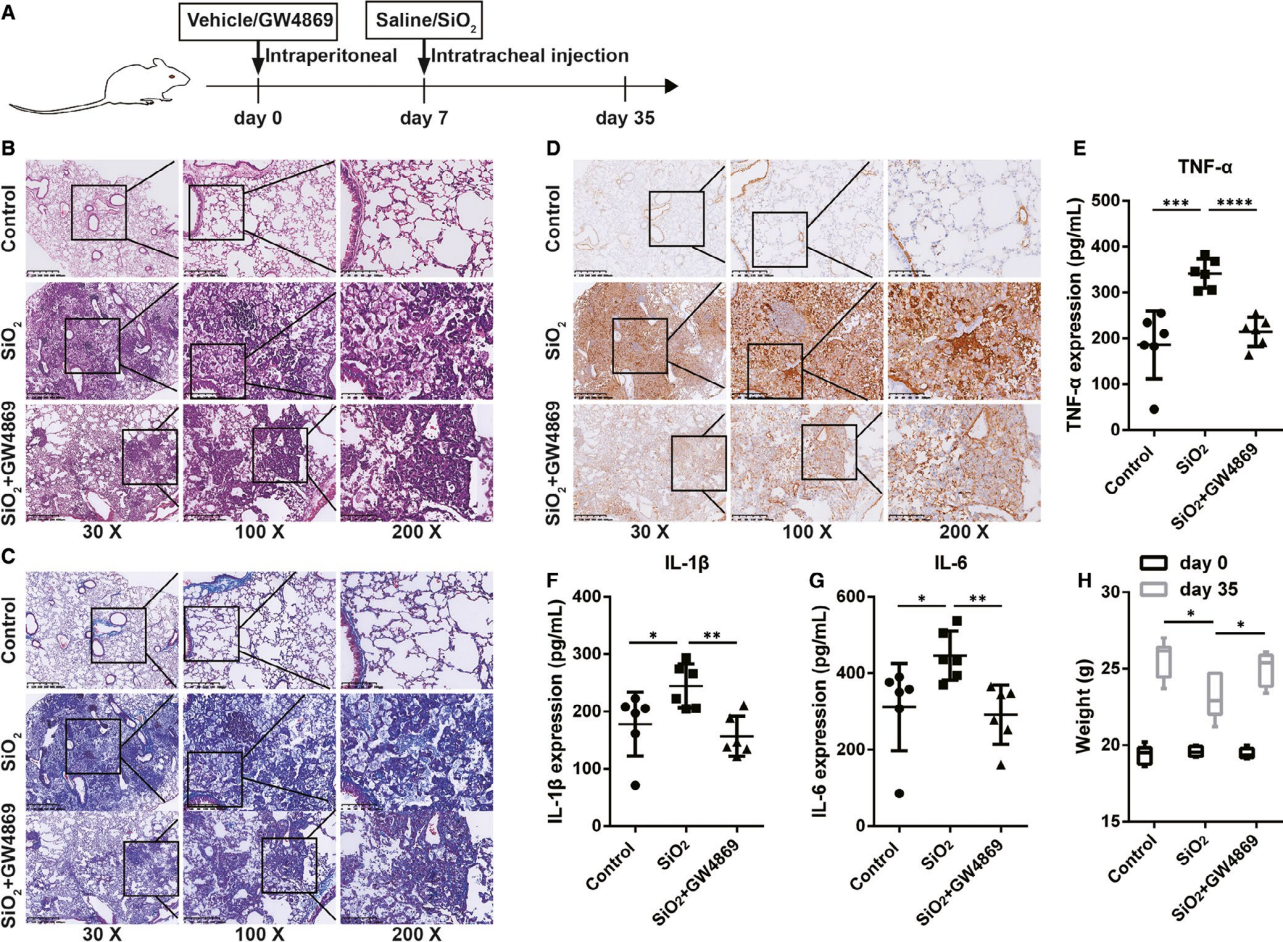
Inhibition of exosome generation reduces proinflammatory cytokine production and lung fibrosis in vivo (A) Schematic of mouse silicosis models. (B) H&E staining was performed on the lung tissues from mice given various treatments (control, SiO_2_, and SiO_2_ + GW4869). The animals were killed on the 28th day after silica exposure. (C) Masson's trichrome staining was performed on the same samples as in B. (D) Immunohistochemical staining identifying the localization of α‐SMA in lung tissues. Magnification: 30×, 100×, and 200×. (E‐G) ELISA analyses of the expression levels of TNF‐α (E), IL‐1β (F) and IL‐6 in BALF. (G). (H) Analysis of weight in each group. n = 6 mice per group. Student's *t* test, *P < .05, **P < .01, ***P < .001

Next, we investigated whether exosomes contributed to the progression of fibrosis in a silicosis model. To determine the exosome‐mediated promotive effects on pulmonary fibrosis in silicosis, GW4869 was used to block the generation of exosomes. We constructed a silicosis pulmonary fibrosis model given various treatments (Figure 4A). Either PBS or GW4869 (2.5 μg/g per day) was used to pre‐treat mice for 7 days prior to silica suspension exposure; the silica suspension (100 mg/kg) was then administered by intrabronchial injection. GW4869 was continuously administered to the mice until they were killed on day 35 (28 days after exposure to the silica suspension), and the BALF and lung tissues of the mice were harvested and examined. We found that, in BALF, the expression levels of HSP70, TSG101 and CD63 were significantly up‐regulated in SiO_2_‐Exos (the SiO_2_ group) compared with control‐Exos (the control group) and down‐regulated in SiO_2_ + GW4869‐Exos (the SiO_2_ + GW4869 group) compared with SiO_2_‐Exos (Figure S1A). Consistent with these observations, the total protein content of exosomes also increased significantly in SiO_2_‐Exos compared with control‐Exos and decreased in SiO_2_+GW4869‐Exos compared with SiO2‐Exos (Figure S1B). These results suggested that treatment with GW4869 could inhibit exosome generation in mice. H&E staining showed that in the control group, the lung tissue had clear and intact alveolar structures without obvious inflammatory cell infiltration or fibrosis. In contrast, in the SiO_2_ group, the alveolar structures were significantly damaged, along with extensive inflammatory cell aggregation and silicon nodule formation (Figure 4B). Masson's trichrome staining demonstrated that compared with the control group, the sizes of dark blue staining regions in the SiO_2_ group were increased significantly (Figure 4C). However, the regions of inflammatory cell aggregation and collagen fibre deposition were reduced accompanied by weaker staining in the SiO_2_ + GW4869 group (a group including mice pre‐treated with GW4869) than in the SiO_2_ group (Figure 4B‐C). Additionally, the protein expression of α‐SMA was markedly up‐regulated in the SiO_2_ group compared with the control group and down‐regulated in the SiO_2_+GW4869 group compared with the SiO_2_ group (Figure 4D). Furthermore, the results of an analysis of proinflammatory cytokines in the BALF revealed significant increases in the levels of TNF‐α (Figure 4E), IL‐1β (Figure 4F) and IL‐6 (Figure 4G) in the SiO_2_ group compared with the control group. Pre‐treatment with GW4869 resulted in a significant decrease in the production of TNF‐α, IL‐1β and IL‐6 in BALF induced by SiO_2_ (Figure ). In addition, we observed that the average bodyweight was decreased in the SiO_2_ group compared with the control group and increased in the SiO_2_+GW4869 group compared with the SiO_2_ group (Figure 4H).

### 3.5 SiO_2_‐Exos regulate myofibroblast activation through ER stress

**FIGURE 5:**
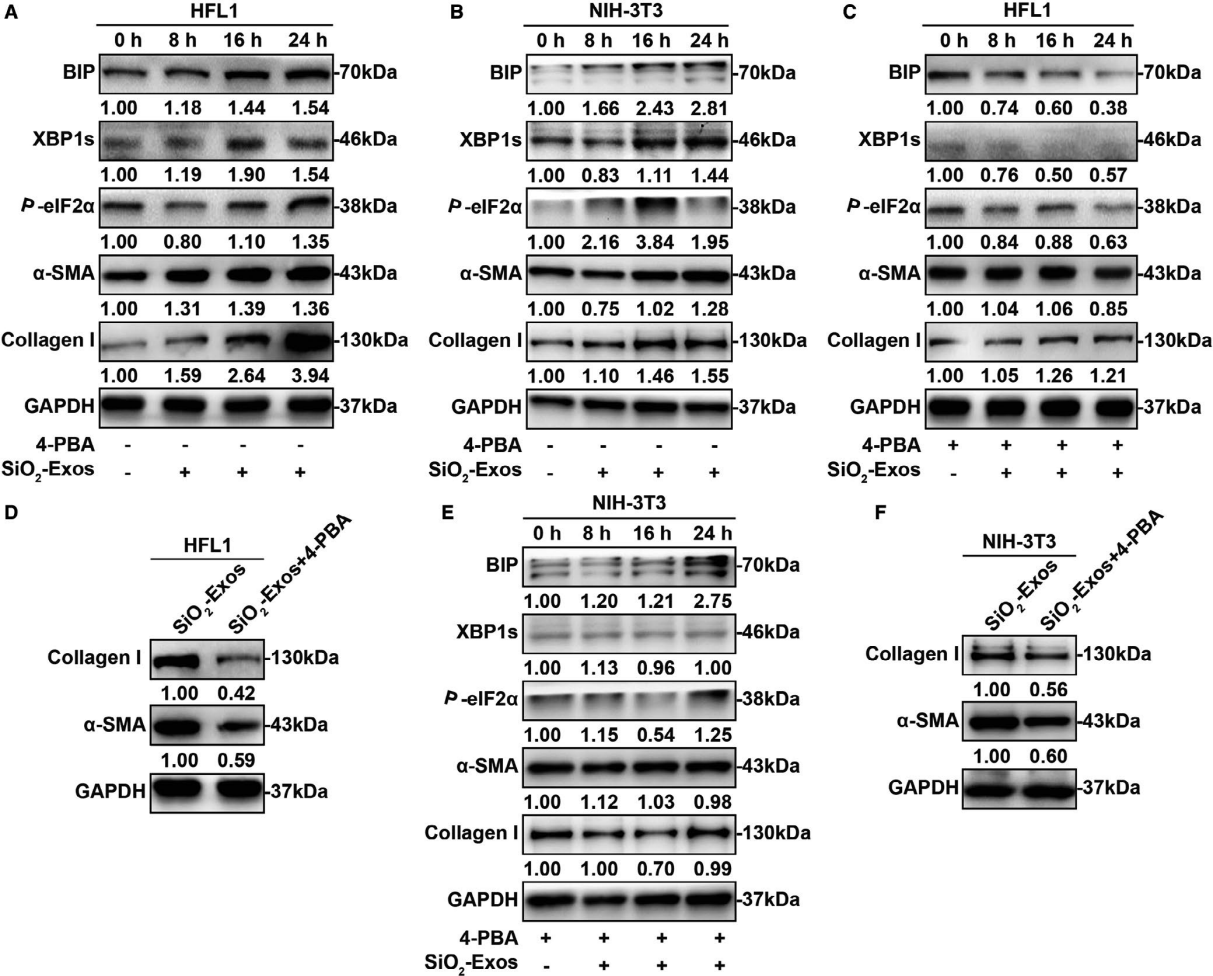
SiO_2_‐Exo‐induced myofibroblast differentiation is ER stress dependent (A‐C, E) Western blot analysis of BIP, XBP1s, *P*‐eIF2α, α‐SMA, Collagen I, and GAPDH in HFL1 cells (A, C) and NIH‐3T3 cells (B, E) incubated with SiO_2_‐Exos (± 4‐PBA) at different points in time (0, 8, 16 or 24 h). (D, F) Analysis of collagen I, α‐SMA, and GAPDH in HFL1 cells (D) and NIH‐3T3 cells (F) incubated with SiO_2_‐Exos or SiO_2_‐Exos +4‐PBA for 24 h

ER stress and the UPR are known to contribute to the development of pulmonary fibrosis by regulating myofibroblast differentiation.^20^, ^21^, ^22^, ^36^ Additionally, recent studies have shown that exosomes modulate apoptosis through ER stress.^37^, ^38^, ^39^ Consequently, we hypothesized that SiO_2_‐Exos may regulate myofibroblast activation through ER stress. First, to investigate whether ER stress is involved in regulating myofibroblast differentiation induced by SiO_2_‐Exos, we examined the expression levels of several protein markers related to these processes. SiO_2_‐Exos (± 4‐PBA) were used to treat fibroblasts, and the expression levels of UPR‐related genes (XBP1s and *P*‐eIF2α), ER stress marker (BIP) and myofibroblast differentiation markers (collagen I and α‐SMA) were measured. These results showed that after treatment with SiO_2_‐Exos, the expression levels of BIP, XBP1s and *P*‐eIF2α were up‐regulated over time (Figure 5A,B). Similarly, the expression of collagen I and α‐SMA in HFL1 cells (Figure 5A) and NIH‐3T3 cells (Figure 5B) was also increased over time. When SiO_2_‐Exos and the ER stress inhibitor 4‐PBA were cocultured with fibroblasts, the expression levels of BIP, XBP1s and *P*‐eIF2α were down‐regulated (Figure 5C,E). Consistent with these observations, the expression of collagen I and α‐SMA in HFL1 cells (Figure 5C,D) and NIH‐3T3 cells (Figure 5E,F) was decreased in the combination group (SiO_2_‐Exos + 4‐PBA) compared with the SiO_2_‐Exos group.

### 3.6 Inhibition of ER stress attenuates fibroblast proliferation and migration induced by SiO_2_‐Exos

**FIGURE 6:**
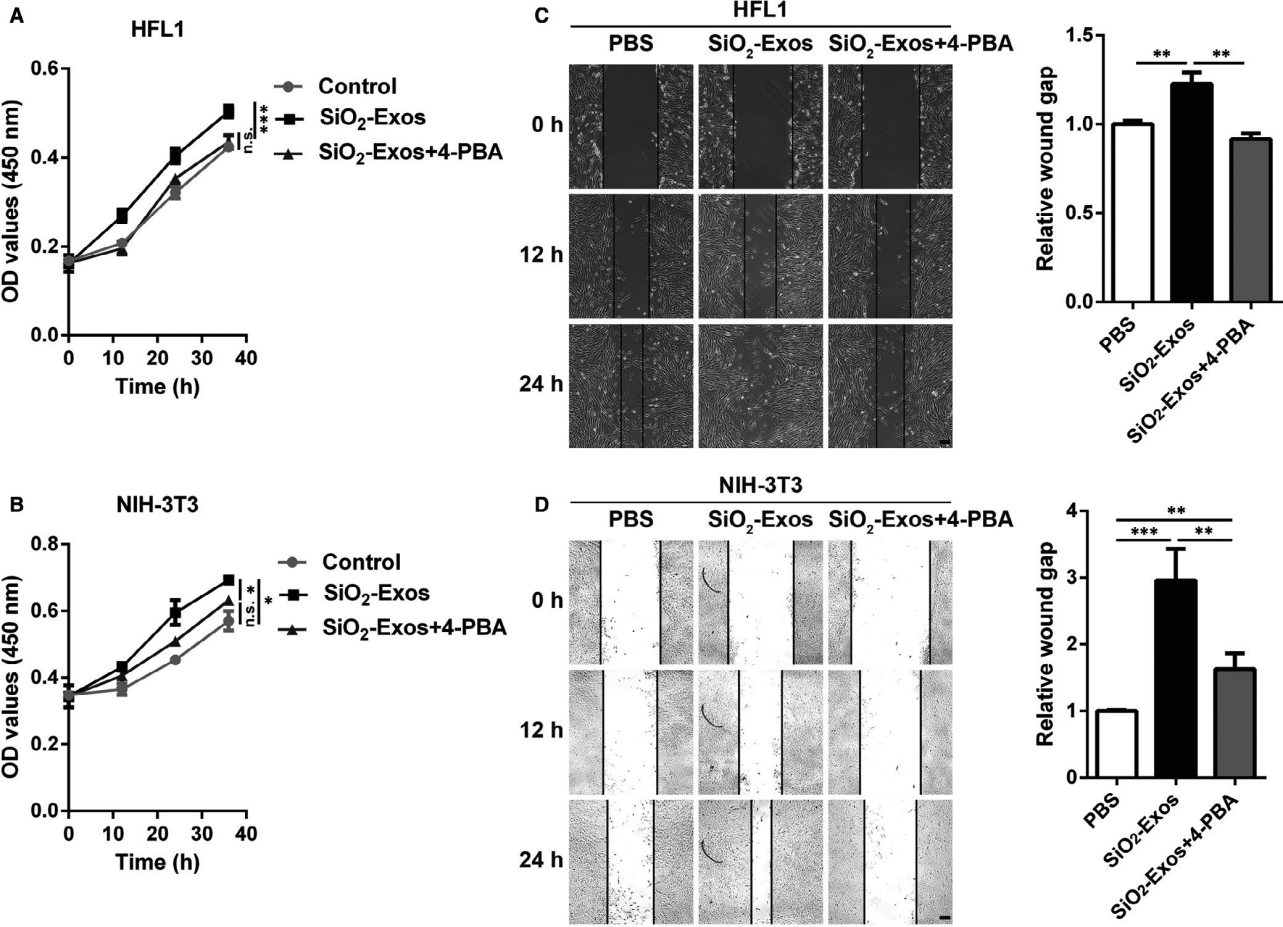
Inhibition of ER stress attenuates SiO_2_‐Exo‐induced lung fibroblast proliferation and migration (A‐B) CCK‐8 assay was used to evaluate the viability of HFL1 cells (A) and NIH‐3T3 cells (B) incubated with SiO_2_‐Exos ± 4‐PBA. Two‐way ANOVA; *P < .05, **P < .01, ***P < .001, n.s: not significant. (C‐D) The migration of HFL1 cells (C) and NIH‐3T3 cells (D) was assessed by wound closure assay. Scale bar: 100 μm. Student's *t* test; *P < .05, **P < .01, ***P < .001, n.s: not significant

We next examined whether ER stress is involved in fibroblast proliferation and migration induced by SiO_2_‐Exos. The fibroblasts were treated with SiO_2_‐Exos (± 4‐PBA), and then, fibroblast proliferation and migration were evaluated. The results showed that SiO_2_‐Exos promoted HFL1 and NIH‐3T3 cell proliferation (Figure 6A,B) and migration (Figure 6C,D), and these facilitating effects were suppressed by 4‐PBA.

## 4 DISCUSSION

Silicosis is a lethal pneumoconiosis and used to be a disease of miners. However, because of poor surveillance and a lack of effective protection in contemporary industrial manufacturing, such as the sand blasting process, silicosis is re‐emerging around the world.^2^ To date, no proven curative treatment for silicosis exists, and treatment options are limited. Hence, it is necessary to further explore the pathogenesis of silicosis and improve the effects of therapies.

After silica dust exposure, alveolar macrophages are the first line of defence and initially activate the inflammatory response by producing various kinds of chemokines, cytotoxic oxidants, proteases and cytokines that stimulate fibroblasts to produce excessive ECM, eventually resulting in lung remodelling and fibrogenesis.^40^, ^41^ However, the cell‐to‐cell communication between macrophages and fibroblasts has not been fully elucidated. Exosomes are secreted membranous nanoparticles and mediators that facilitate communication with other cells and tissues.^42^, ^43^ Increasing exosome secretion may be a danger signal in disease.^29^ Aina Martin‐Medina et al ^17^ found that the release of exosomes into the BALF was significantly increased in IPF patients and mice after intratracheal bleomycin administration. Exosomes are intercellular carriers of specific contents that depend on the cellular context and source and play essential roles in the biological processes of cells and diseases.^15^, ^16^, ^17^ Guozhen Wang et al ^43^ identified 5 056 proteins in macrophage‐exosomes. Compared with control‐exosomes, LPS‐treated macrophage‐derived exosomes included 341 proteins with increased levels and 363 proteins with reduced levels, including proinflammatory cytokines and chemokines such as CXCL2, CCL22, TNF and CCL3. Previous studies indicate that exosomes are widely involved in the physiological and pathological processes of chronic lung diseases.^15^, ^16^, ^44^ Aina Martin‐Medina et al ^17^ reported that exosomes from the BALF of IPF patients increased lung fibroblast proliferation. However, the release level and function of macrophage‐derived exosomes in the pathogenesis of silicosis remain largely unknown. Hence, we hypothesized that macrophage‐derived exosomes may play a part in the pathological processes of silicosis. Our study demonstrated that, in vitro, SiO_2_ could induce a significant increase in exosome secretion by macrophages and that SiO_2_‐Exos could effectively promote myofibroblast differentiation, proliferation and migration. In vivo, inhibition of exosome generation by using GW4869 dampened SiO_2_‐induced pulmonary fibrosis and inflammation. In addition, recent studies have demonstrated that exosomes regulate cellular biological behaviour and disease through ER stress.^45^, ^46^, ^47^ ER stress‐induced XBP1s can be incorporated into exosomes and transmitted intercellular.^46^ Wu C H et al ^47^ reported that exosomes derived from bladder cancer triggered tumorigenesis in non‐malignant cells by inducing the UPR. Yu Y et al ^48^ demonstrated that endotheliocyte‐derived exosomes attenuated neuronal apoptosis and inflammation by inhibiting ER stress. ER stress is widely involved in myofibroblast activation and EMT in pulmonary fibrosis.^25^, ^49^ Consequently, we hypothesized that ER stress may contribute to SiO_2_‐Exo‐induced myofibroblast activation. Our study revealed that fibroblast activation induced by SiO_2_‐Exos was dependent on ER stress activation. However, we have not yet determined which of the molecular and cellular cargo components in exosomes contribute to the fibrotic pathomechanism in silicosis. Moreover, the mechanism of ER stress activation induced by SiO_2_‐Exos will also be the focus of our further research.

In this study, we demonstrate that SiO_2_‐Exos are profibrogenic and contribute to pulmonary fibrosis and inflammation during silicosis. Inhibition of exosome generation can dampen pulmonary inflammation and attenuate pulmonary fibrosis. In addition, recent studies have shown that exosomes have great potential in the treatment of pulmonary fibrosis.^50^, ^51^, ^52^ Therefore, macrophage‐derived exosome may be a therapeutic target for silicosis.

## CONFLICT OF INTEREST

The authors declare that there is no conflict of interest that could be perceived as prejudicing the impartiality of the research reported.

## AUTHOR CONTRIBUTIONS

**Xiaofeng Qin:** Investigation (equal); Writing‐original draft (equal). **Xiaofang Lin:** Investigation (equal); Methodology (equal). **Lang Liu:** Investigation (equal). **Ying Li:** Investigation (equal). **Xiang Li:** Investigation (equal). **Zhenghao Deng:** Investigation (equal). **Huiping Chen:** Investigation (equal). **Hui Chen:** Investigation (equal). **Zhiyuan Niu:** Investigation (equal). **Zisheng Li:** Investigation (equal). **Yongbin Hu:** Conceptualization (lead); Funding acquisition (supporting); Supervision (lead); Writing‐original draft (equal).

## Supporting information

Figure S1

## Data Availability

The data that support the findings of this study are included within the article.
