# Supplementary material for: Macrophage‐derived exosomes mediate silica‐induced pulmonary fibrosis by activating fibroblast in an endoplasmic reticulum stress‐dependent manner
Source: J Cell Mol Med. 2021 Apr 8;25(9):4466–77. doi: 10.1111/jcmm.16524 (PMC8093963; doi:10.1111/jcmm.16524)
Supplement: Supplementary file 1 — Figure S1 [file JCMM-25-4466-s001.pdf]

SUPPLEMENTAL MATERIAL

Supplemental figure and supplemental figure legend

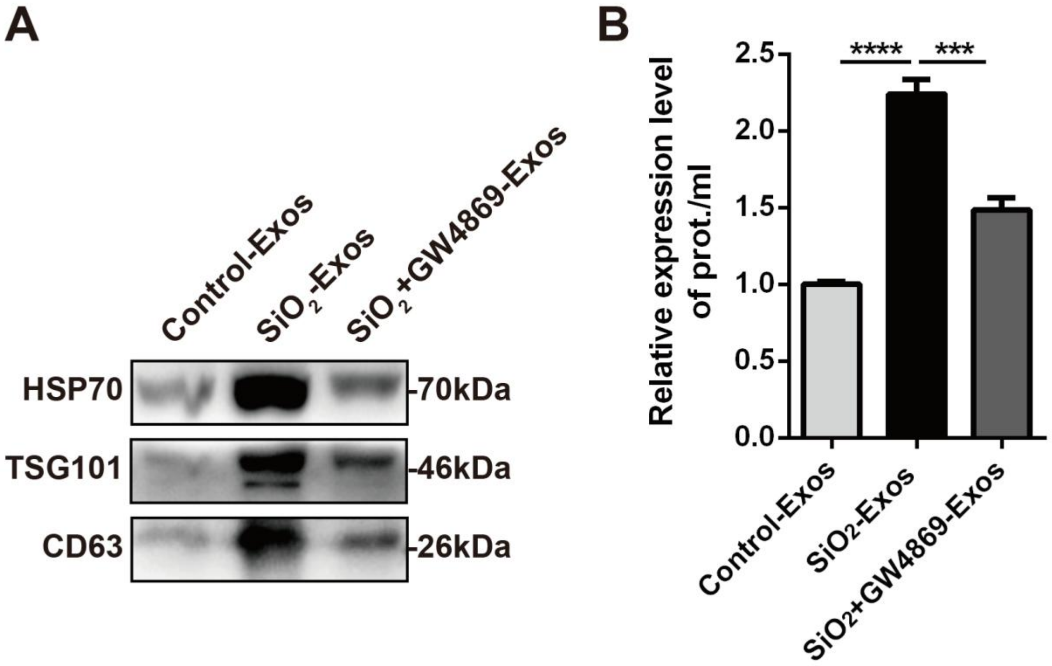

SUPPLEMENTAL FIGURE 1 Exosome secretion in bronchoalveolar lavage fluid (BALF) of mice treated with Nacl, SiO_2, or SiO_2+GW4869 (A) The expression levels of the exosome-related markers HSP70, TSG101 and CD63 were detected by Western blot analysis. Exosomes were isolated from the BALF of Nacl-treated (Control-Exos), SiO_2-treated (SiO_2-Exos) or SiO_2+GW4869-treated (SiO_2+GW4869-Exos) mice after SiO_2 exposure for 28 days. (B) Quantification of the total protein in exosomes isolated from the BALF of Nacl-treated (Control-Exos), SiO_2-treated (SiO_2-Exos) or SiO_2+GW4869-treated (SiO_2+GW4869-Exos) mice after SiO_2 exposure for 28 days. n = 8 mice per group. Student's *t* test; ***$p < 0.001$
